# Predicting major bleeding among hospitalized patients using oral anticoagulants for atrial fibrillation after discharge

**DOI:** 10.1371/journal.pone.0246691

**Published:** 2021-03-03

**Authors:** Jakub Z. Qazi, Mireille E. Schnitzer, Robert Côté, Marie-Josée Martel, Marc Dorais, Sylvie Perreault

**Affiliations:** 1 Faculty of Pharmacy, University of Montreal, Montreal, Quebec, Canada; 2 School of Public Health, University of Montreal, Montreal, Quebec, Canada; 3 StatSciences Inc., Notre-Dame-de-l’Île-Perrot, Quebec, Canada; Karolinska Institutet, SWEDEN

## Abstract

**Aim:**

Real-world predictors of major bleeding (MB) have been well-studied among warfarin users, but not among all direct oral anticoagulant (DOAC) users diagnosed with atrial fibrillation (AF). Thus, our goal was to build a predictive model of MB for new users of all oral anticoagulants (OAC) with AF.

**Methods:**

We identified patients hospitalized for any cause and discharged alive in the community from 2011 to 2017 with a primary or secondary diagnosis of AF in Quebec’s RAMQ and Med-Echo administrative databases. Cohort entry occurred at the first OAC claim. Patients were categorized according to OAC type. Outcomes were incident MB, gastrointestinal bleeding (GIB), non-GI extracranial bleeding (NGIB) and intracranial bleeding within 1 year of follow-up. Covariates included age, sex, co-morbidities (within 3 years before cohort entry) and medication use (within 2 weeks before cohort entry). We used logistic-LASSO and adaptive logistic-LASSO regressions to identify MB predictors among OAC users. Discrimination and calibration were assessed for each model and a global model was selected. Subgroup analyses were performed for MB subtypes and OAC types.

**Results:**

Our cohort consisted of 14,741 warfarin, 3,722 dabigatran, 6,722 rivaroxaban and 11,196 apixaban users aged 70–86 years old. The important MB predictors were age, prior MB and liver disease with ORs ranging from 1.37–1.64. The final model had a c-statistic of 0.63 (95% CI 0.60–0.65) with adequate calibration. The GIB and NGIB models had similar c-statistics of 0.65 (95% CI 0.63–0.66) and 0.67 (95% CI 0.64–0.70), respectively.

**Conclusions:**

MB and MB subtype predictors were similar among DOAC and warfarin users. The predictors selected by our models and their discriminative potential are concordant with published data. Thus, these models can be useful tools for future pharmacoepidemiologic studies involving older oral anticoagulant users with AF.

## Introduction

Atrial fibrillation (AF) is the most common cardiac arrhythmia worldwide with increasing incidence due to the aging population [1–3]. It is associated with 5-fold and 3-fold increases in the risk of stroke and systemic embolism, respectively, with AF-associated stroke showing twice the risk of thirty-day all-cause mortality relative to non-AF associated stroke [4–6]. Before 2010, the vitamin K antagonist, warfarin, was the only medication used for stroke and systemic embolism prevention for AF patients at moderate and high risk of these outcomes [7–9]. However, warfarin is associated with a high risk of major bleeding (MB; 7.2 per 100 person-years), of which the most common type is gastrointestinal bleeding (GIB) and the most lethal type, intracranial hemorrhage (ICH) [9, 10]. In 2010, the first of the direct oral anticoagulants (DOAC) received approval from the US Food and Drug Administration for stroke prevention in patients diagnosed with atrial fibrillation (AF). In addition to circumventing the need for INR, the DOACs (dabigatran, rivaroxaban, apixaban and edoxaban) presented pharmacokinetic, pharmacodynamic and safety advantages over warfarin [9].

The four DOAC clinical trials for AF, namely RE-LY, ROCKET-AF, ARISTOTLE and ENGAGE-AF, concluded non-inferior (or superior, in the case of ARISTOTLE) efficacy in reducing stroke, systemic embolism and all-cause mortality rates for each DOAC relative to warfarin and a lower risk of MB for all DOACs [11–16]. Given that randomized clinical trials (RCTs) do not account for real-world patient characteristics, pharmacoepidemiologic studies were required to complement and confirm RCT findings. According to meta-analyses of observational studies, DOAC effectiveness and safety with respect to MB risk was equivalent to warfarin’s [17, 18]. Additionally, pooled DOAC analyses were associated with a greater GIB risk and lower ICH risk in patients over 75 years old [17, 18]. However, apixaban was the only DOAC with an associated lower risk of MB, GIB, and ICH relative to warfarin. It also had an associated lower risk of MB relative to the other DOACs [17, 19, 20]. Within each DOAC subgroup, significant heterogeneity existed in at least one of the bleeding outcomes (MB, ICH or GIB) [17, 21, 22].

To ensure oral anticoagulant (OAC) safety, the risk-benefit profile needs to be carefully assessed while taking into account factors associated with a predisposition to bleeding [9]. The HAS-BLED, a scoring system used to identify patients at risk of bleeding, was developed based on warfarin user data and validated among rivaroxaban users [23, 24]. Since then, other MB prediction scores have been developed to improve bleeding prediction within this population. The HEMORR_2_AGES and ATRIA scores were derived from warfarin user data, while the ORBIT-AF also accounted for dabigatran user data. Ultimately, the ABS score was derived from DOAC and warfarin user data [9, 25–28]. However, given that the HAS-BLED is still the most commonly used score, a user-friendly MB prediction tool derived from a recent population of OAC users is essential.

Moreover, the HAS-BLED and other prediction models were developed to predict any MB, but it is also of interest to establish risk factors for specific MB subtypes, GIB, non-GI extracranial bleeding (NGIB) and ICH [9, 25–28]. The lack of prediction models for MB subtypes, and the lack of studies identifying MB subtype-specific predictors makes it difficult to accurately monitor MB and actively engage in their prevention [29, 30]. Specifically, we aimed to develop predictive models for MB and for the most prevalent MB subtypes (GIB and NGIB) based on data from real-world patients with AF taking any type of OAC. Therefore, our primary objective is to establish a model to predict MB in a population of all OAC users with AF. Our second objective is to identify important predictors of the most prevalent MB subtypes (GIB and NGIB). Our third objective is to compare the predictors of MB between warfarin and DOAC users as well as doing so with the MB subtypes. Our final objective is to evaluate the discriminative potential of the MB model fit to all OAC users for GIB and NGIB.

## Methods

### Data source

Administrative databases have proven to be a widely available and useful tool for pharmacoepidemiologic studies [31, 32]. The data for our study were compiled from a subset of the Régie de l’Assurance Maladie du Québec (RAMQ) drug and medical services database linked to the Med-Echo hospitalization database using encrypted patient healthcare insurance numbers [31, 33–36]. Quebec prescription and hospitalization data have been shown to have a high degree of completeness (with only 0 to 0.4% of data that was missing) and accuracy [31]. Thus, our cohort did not have any missing data.

### Population-based cohort definition

We conducted a cohort study using drug claims and diagnostic coding data from the Quebec RAMQ and Med-Echo administrative databases. We identified adult patients who were hospitalized for all cause and discharged alive in the community from January 1, 2011 to December 31, 2017 with a primary or a secondary diagnosis of AF. They were identified using ICD-9 (427.3, 427.31 or 427.32) or ICD-10 (I48) codes [37, 38]. For patients with more than one admission with an AF diagnosis, we used the first date of admission. The ICD-9 codes displayed median positive predictive values of 89% and 95.7% in two distinct validation studies [37, 38].

**Fig 1:**
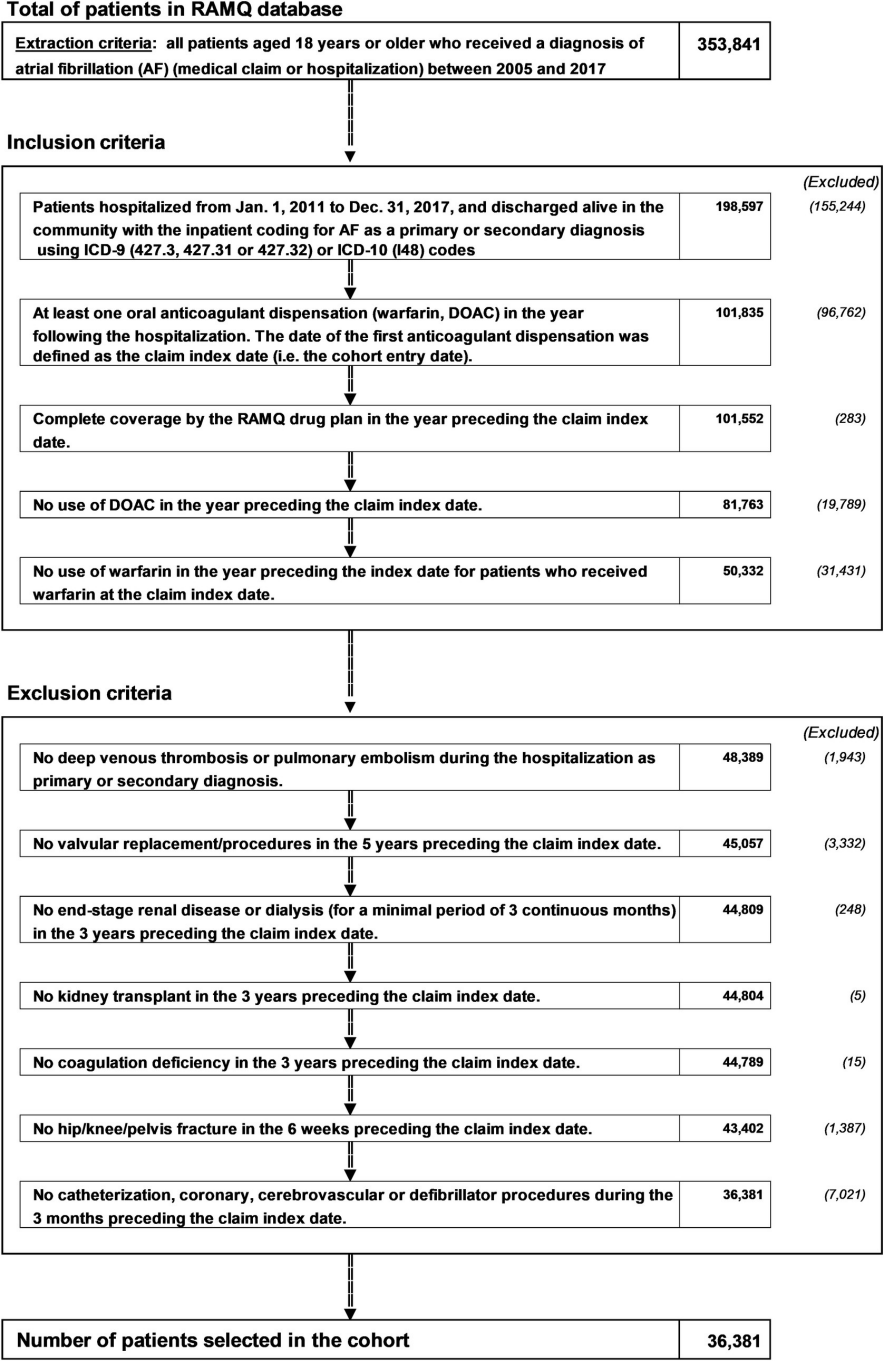
Population-based cohort definition flowchart. AF: atrial fibrillation; OAC: oral anticoagulant, DOAC: direct oral anticoagulant, RAMQ: Régie d’Assurance-Maladie du Québec.

Patients included in the cohort had to have a filled prescription of at least one of the DOACs (dabigatran, rivaroxaban and apixaban) or warfarin in the year following hospitalization, but could not have used any OAC one year prior to this claim. For this reason, they also had to have continuous RAMQ drug plan coverage for at least one year prior to cohort entry (see Fig 1). The date of cohort entry (or study index) was defined as the first filled OAC prescription after hospital discharge.

We excluded patients with OAC contraindications (end‐stage chronic renal disease [ESRD] or dialysis for a minimum of 3 months) followed by kidney transplantation within 3 years before cohort entry. We also excluded patients with a non-AF indication for DOAC anticoagulation such as post-orthopedic surgery (hip or knee replacement 6 weeks before cohort entry) and a diagnosis of venous thromboembolism (defined as either deep vein thrombosis or pulmonary embolism) during the hospitalization period. Finally, we excluded those having undergone cardiac valve replacement up to 5 years prior to cohort entry.

### Oral anticoagulant exposure

OAC exposure was defined as filing a new claim for warfarin or a DOAC (all dosages approved in Canada included) after hospital discharge. Given that the database had very few users of edoxaban, these patients were not included in our cohort. Patient treatment initiation was determined using dispensation dates of the OAC prescriptions. All individuals were new users, i.e., individuals who had not been exposed to any OAC at least one year prior to cohort entry.

### Study outcomes

The primary outcomes were MB including GIB, NGIB and ICH. MB, GIB, NGIB and ICH were defined as the first instance of each respective bleeding event leading to a hospitalization during follow-up and identified using ICD-9 and ICD-10 codes from inpatient claims (S1 Table). These outcomes were defined using 6 distinct observational studies [39–45]. When multiple of either MB subtypes occurred, only the first of that respective MB subtype was evaluated as the primary outcome (e.g. GIB was defined as the first GIB during the follow-up period). These codes have been externally validated with positive predictive value ranging from 85% to 95% [46–48]. Patient follow-up began from the first OAC claim until the earliest occurrence of one of the following events: MB event, end of coverage of the RAMQ drug insurance, date of death, 1 year of follow-up or end of the study.

### Baseline characteristics and predictor candidates

Sociodemographic variables (age, sex, and material and social deprivation indices) were defined at cohort entry [49]. Associated morbidities were assessed up to 3 years prior to cohort entry. They included stroke/transient ischemic attack, hypertension, dyslipidemia, cardiomyopathy, coronary artery disease, acute myocardial infarction, peripheral vascular disease (PVD), chronic heart failure, anemia, chronic kidney disease (CKD), severe kidney disease (creatinine clearance < 30 ml /min), acute renal failure, liver disease, diabetes mellitus, asthma and chronic obstructive pulmonary disease (COPD), history of MB, and prior Helicobacter *Pylori* infection [40, 50, 51]. The CHA_2_DS_2_-VASc score (stroke risk), a modified HAS-BLED (bleeding risk) excluding labile INR, and the Charlson-Deyo comorbidity index, were assessed up to 3-years prior to cohort entry (S2 and S3 Tables for coding algorithms). Finally, we documented baseline medication use, which included antiplatelets, proton pump inhibitors (PPIs), non-steroidal anti-inflammatory agents (NSAIDs), digoxin, amiodarone, antidepressants, β-blockers, calcium channel blockers, inhibitors of renin-angiotensin system, diuretics, loop diuretics, antidiabetics up to 2 weeks prior to cohort entry.

### Statistical analyses

First, we generated descriptive data for warfarin, DOAC and OAC new users with and without GIB, NGIB and MB. We calculated percentages for binary and categorical variables and means with standard deviations for continuous ones.

We determined the cumulative incidence of MB, GIB, NGIB and ICH (events per 100 person-years), respectively. We then generated Kaplan-Meier curves for each dose-stratified OAC treatment group to assess cumulative MB, GIB and NGIB incidences within the first year after cohort entry. We used the log rank test to compare each of the MB, GIB and NGIB cumulative incidences of each DOAC treatment group to those of warfarin users.

We selected candidate variables to be evaluated as predictors of any MB or MB subtypes based on availability in our dataset and clinical relevance, which was defined as inclusion in bleeding scores, significant differences in baseline measurements, or a strong association with MB based on narrative review [25, 29, 52]. We used the Least Absolute Shrinkage and Selection Operator (LASSO) method, which introduces a penalty/bias to each coefficient of a regression model to select relevant predictors and to minimize overfitting, and the adaptive LASSO (adaLASSO), which uses the same principle while applying a larger penalty to smaller coefficients than to larger ones [53, 54].

Both LASSO and adaLASSO penalties can be incorporated into logistic regression (logistic-LASSO and logistic-adaLASSO, respectively), which perform well when the true model is sparse [53, 54]. Given that the 10 events per predictor rule, proposed to be too conservative for penalty-based regression, was respected for each outcome in the OAC models, we deemed the sample size of this cohort to be sufficiently large to derive robust prediction models (S4 Table) [55]. Most notably, all available data were used to maximize the power and generalizability of the results.

For each outcome, we calculated odds ratios (ORs) for each covariate for the warfarin, DOAC and OAC treatment groups using logistic-LASSO and logistic-adaLASSO regressions (R v3.6.2, package “glmnet”). We did not include 95% confidence intervals (CIs) as it is challenging to interpret them in log-LASSO and log-adaLASSO modelling. We calculated cross-validated concordance statistics (c-statistics) and their 95% CIs using the area under Receiving Operator Curves (auROC) to determine model discrimination (R v3.6.2, package cvAUC) [56]. Finally, the calibration of each model was quantitatively and qualitatively characterized using Hosmer-Lemeshow tests, a chi-squared test of mean squared differences of true and predicted outcomes between quantiles of outcome measurements, and their corresponding calibration plots (R v3.6.2, packages “generalhoslem” and “PredictABEL”) [56]. We then identified the best model, defined as having the best discrimination value, adequate calibration and having selected the least variables within each OAC subgroup (warfarin, DOAC and OAC). Ultimately, we evaluated the final MB model’s performance and evaluated its ability to detect MB subtypes (GIB and NGIB) via discrimination and calibration testing using the previously discussed methods.

### Ethics statement

The protocol was approved by the University of Montreal Health Research Ethics Committee (cert. 17-068-CERESD) and the Committee of Access to Personal Information (CAI).

## Results

### Demographic and clinical characteristics

The cohort of OAC new users diagnosed with AF that have met all inclusion and exclusion criteria comprised of 36,381 patients. The two treatment subgroups consisted of warfarin users (n = 14,741) and DOAC users (n = 21,640). The mean age of patients who experienced bleeding during follow-up and those that did not ranged from 78.9 to 80.9 years old as shown in Table 1. Whether or not they experienced MB, OAC users were more likely to be over the age of 75 (68.3% to 77.4%), had numerous comorbidities (Charlson-Deyo co-morbidity scores from 4.5±3.4 to 5.9±3.9), had a high stroke risk (CHA_2_DS_2_-VASc scores from 3.7±1.4 to 4.0±1.3) and had a high bleeding risk (HAS-BLED scores from 3.1±1.3 to 3.5±1.3), as shown in Table 1. Patients who experienced MB within the year of follow-up were more likely to be over 75 years old (76.1%), had over 5 comorbidities on average (Charlson-Deyo score: 5.3 ± 3.6), a high bleeding risk (HAS-BLED: 3.4 ± 1.2) and a high stroke risk (CHA_2_DS_2_-VASc: 4.0 ± 1.3). Warfarin and DOAC users had a total of 499 and 528 MB events, respectively (Table 1; S5 and S6 Tables).

**Table 1:** Baseline characteristics of OAC new user with and without major bleed in the year of follow-up from 2011 to 2018.

	No major bleeding (n = 35,354)	GI bleeding (n = 438)	Non-GI extracranial bleeding ^a^ (n = 363)	All major bleeding ^b^ (n = 1,027)
**Sociodemographics**				
Age (mean ± SD)	78.9 ± 9.4	80.6 ± 8.0	80.2 ± 8.2	80.9 ± 8.2
Age (%) ^d^				
≥ 75	68.3%	77.4%	72.7%	76.1%
Male (%)	45.9%	45.4%	52.9%	49.1%
Pampalon index elevated social deprivation (%)	26.6%	26.6%	26.5%	26.6%
Pampalon index elevated material deprivation (%)	25.8%	25.8%	25.8%	25.8%
**CHA**_**2**_**DS**_**2**_**-VASc Score (mean ± SD)**	3.7 ± 1.4	4.0 ± 1.3	4.0 ± 1.4	4.0 ± 1.3
**CHA**_**2**_**DS**_**2**_**-VASc Score (%)** ^d^				
0–1	5.9%	2.3%	2.5%	2.3%
2–3	37.7%	31.7%	32.5%	32.3%
4	29.0%	33.8%	31.7%	32.6%
≥ 5	27.4%	32.2%	33.3%	32.7%
**HAS-BLED score (mean ± SD)**	3.1 ± 1.3	3.3 ± 1.2	3.5 ± 1.3	3.4 ± 1.2
**HAS-BLED score (%)** ^d^				
< 3	34.5%	24.2%	22.0%	23.7%
≥ 3	65.5%	75.8%	78.0%	77.3%
**Co-morbidities within 3 years before cohort entry**				
Hypertension	81.6%	87.7%	86.8%	86.6%
Coronary artery disease (excl. MI)	56.0%	51.4%	58.4%	53.9%
Acute myocardial infarction	12.9%	16.0%	23.4%	17.8%
Chronic heart failure	37.4%	47.5%	45.6%	45.9%
Cardiomyopathy	6.2%	6.2%	13.0%	8.3%
Other dysrhythmias	19.8%	17.8%	20.7%	20.1%
Valvular heart disease	18.7%	24.0%	26.5%	23.4%
Stroke/TIA	19.0%	16.2%	19.3%	20.0%
Peripheral vascular (arterial) disease	20.9%	26.7%	31.7%	28.6%
Dyslipidemia	52.2%	56.4%	58.7%	56.7%
Diabetes	34.7%	40.2%	48.5%	42.5%
History of major bleeding ^c^	29.0%	43.6%	47.7%	42.9%
History of intracranial bleeding	3.8%	2.5%	4.4%	5.0%
History of GI bleeding	7.4%	19.0%	11.9%	13.8%
History of other bleeding ^a^	21.8%	32.7%	39.4%	32.5%
Chronic renal failure	35.1%	39.7%	49.0%	42.4%
Chronic renal failure ≤ 30 mL/min	0.5%	0.7%	1.1%	0.8%
Acute renal failure	22.3%	26.7%	34.4%	28.4%
Liver disease	2.1%	5.7%	3.6%	4.0%
Chronic obstructive pulmonary disease/asthma	36.5%	47.0%	49.0%	43.1%
Infection par Helicobacter pylori	0.7%	0.9%	1.4%	0.9%
Depression	11.3%	10.3%	12.7%	13.4%
**Concomitant medication use (within 2 weeks before cohort entry) (%)**				
Statin	44.7%	48.2%	54.3%	51.0%
All antiplatelets ^c^	29.6%	39.0%	40.2%	39.1%
Low dose aspirin (ASA)	26.4%	35.4%	35.8%	35.2%
Oth. antiplatelets (without ASA)	4.8%	6.9%	7.2%	6.3%
Proton pump inhibitors (PPIs)	45.8%	48.2%	56.2%	50.1%
NSAIDs	1.4%	1.1%	0.6%	1.2%
Digoxin	11.6%	12.3%	13.2%	12.6%
Amiodarone	8.7%	8.5%	11.6%	9.8%
Antidepressants	16.5%	18.5%	20.1%	20.2%
B-Blockers	62.9%	58.2%	59.2%	60.5%
Calcium channel blockers	37.3%	39.5%	36.4%	38.6%
Inhibitors of renin-angiotensin system	36.8%	36.5%	42.4%	39.8%
Diuretics	38.4%	45.4%	49.9%	45.4%
Loop diuretics	31.2%	38.6%	41.6%	37.8%
Antidiabetics	20.4%	24.0%	30.3%	26.2%
**OAC type at cohort entry**				
Warfarin	40.3%	46.6%	46.0%	48.6%
Dabigatran 110 mg	6.2%	8.5%	6.9%	7.8%
Dabigatran 150 mg	4.1%	3.4%	2.5%	2.9%
Rivaroxaban 15 mg	5.0%	6.6%	8.0%	6.7%
Rivaroxaban 20 mg	13.5%	12.6%	14.3%	11.6%
Apixaban 2.5 mg	11.4%	8.9%	7.7%	8.9%
Apixaban 5 mg	19.6%	13.5%	14.6%	13.5%
**Charlson score (mean ± SD)**	4.5 ± 3.4	5.2 ± 3.4	5.9 ± 3.9	5.3 ± 3.6
**Charlson score** < 4 (%) ^d^	45.7%	36.1%	29.2%	34.5%
**Charlson score** ≥ 4 (%) ^d^	54.3%	63.9%	70.8%	65.5%

^a^Non-GI extracranial major bleeding as an outcome or a predictor includes vitreous, urogenital, hemoperitoneal and unspecified major bleeding as well as hemoarthrosis, hemopericardium, hemoptysis, hematuria and post-bleeding anemia.

^b^All major bleedings included GI, non-GI extracranial major bleeding and intracranial bleeding.

^c^Represents a history of at least one of the bleeding subcategories OR at least one prescription of antiplatelet subcategory. Although each subcategory is mutually exclusive, the totals will not add up to the parent variable.

^d^Each categorization is clinically justifiable. A HAS-BLED≥3 implies high bleeding risk, a CHA₂DS₂-VASc≥2 implies high stroke risk and an age≥75 guarantees oral anticoagulation in accordance to AF guidelines. Lastly, a Charlson score cut-off of 4 was chosen since it was close to the lowest average value for any of the subgroups.

### Treatment-specific cumulative incidence measurements

**Fig 2:**
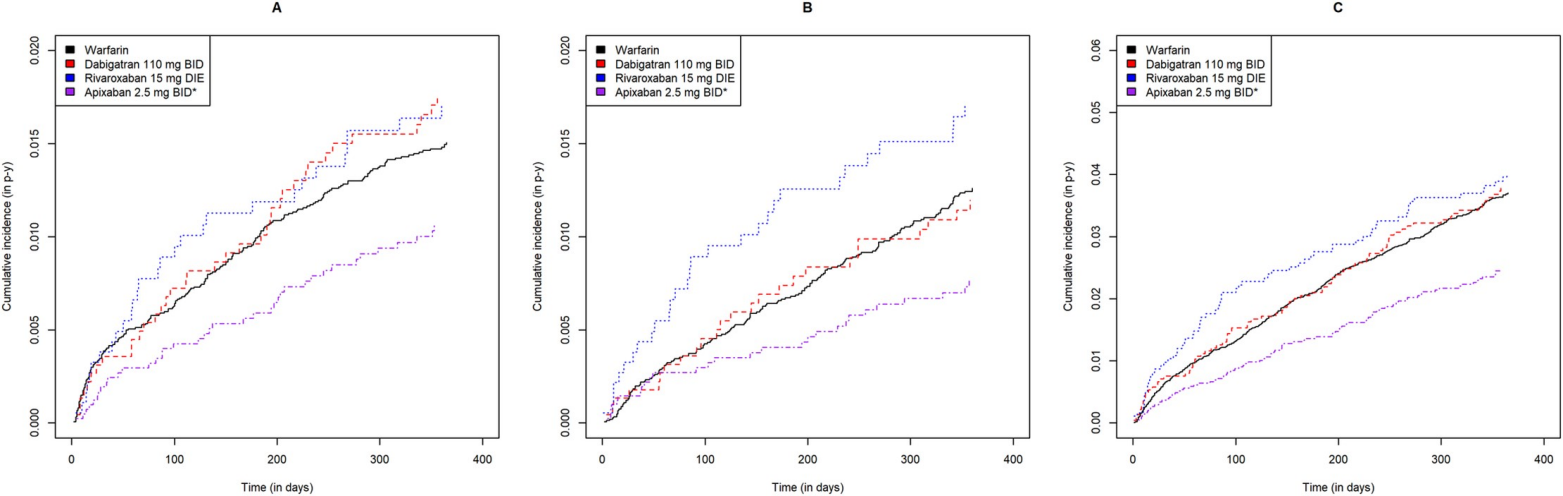
Gastrointestinal, non-gastrointestinal extracranial and all major bleeding cumulative incidence curves for each direct oral anticoagulant at low dose relative to warfarin. Warfarin, dabigatran, rivaroxaban and apixaban are shown in black, red, blue and purple, respectively. Gastrointestinal, non-gastrointestinal and all major bleeding are shown from left to right. * statistically significant difference relative to warfarin (p<0.05).

**Fig 3:**
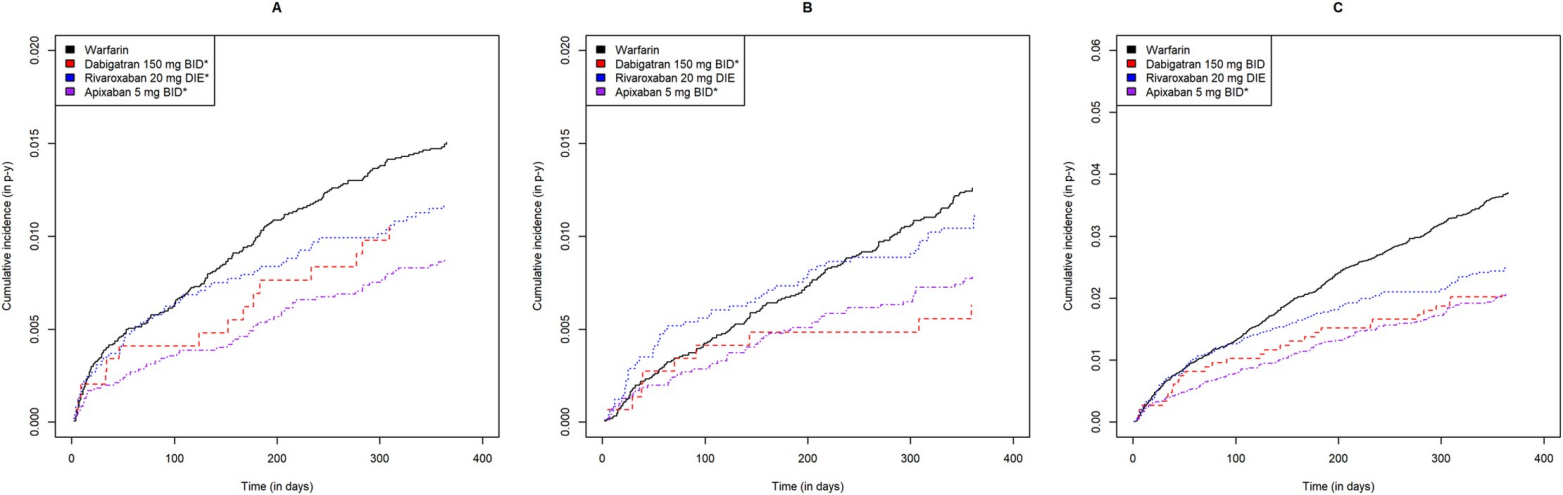
Gastrointestinal, non-gastrointestinal extracranial and all major bleeding cumulative incidence curves for each direct oral anticoagulant at high dose relative to warfarin. Warfarin, dabigatran, rivaroxaban and apixaban are shown in black, red, blue and purple, respectively. Gastrointestinal, non-gastrointestinal and all major bleeding are shown from left to right. * statistically significant difference relative to warfarin (p<0.05).

Including both approved dosages, DOAC users had cumulative ICH, GIB, NGIB, and MB incidences ranging from 0.35 to 0.92, 0.89 to 1.80, 0.64 to 1.77 and 2.11 to 4.27 events per 100 person-years, respectively (Table 2). Warfarin users had cumulative ICH, GIB, NGIB and MB incidences of 1.05, 1.57, 1.28 and 2.84 events per 100 person-years, respectively (Table 2). As shown in Figs 2 and 3, apixaban users had lower incidences of all bleeding subtypes relative to warfarin users for both dosages (log rank p<0.05).

**Table 2:** Crude cumulative incidence of all major bleeds among warfarin, low dose and high dose OAC users with each major bleeding subtype one year after cohort entry between 2011 and 2018.

	Warfarin DIE (n = 14,741)	Dabigatran 110 mg BID (n = 2,255)	Dabigatran 150 mg BID (n = 1,467)	Rivaroxaban 15 mg DIE (n = 1,846)	Rivaroxaban 20 mg DIE (n = 4,876)	Apixaban 2.5 mg BID (n = 4,127)	Apixaban 5 mg BID (n = 7,069)
**Major gastrointestinal bleeding**							
Number with bleeds	204	37	15	29	55	39	59
Total person-years	13,021.8	2,049.9	1,404.6	1,618.8	4,565.5	3,566.4	6,606.5
Rate of bleed (per 100 person-years) ^b^	1.57 (1.36–1.79)	1.80 (1.28–2.44)	1.01 (0.61–1.70)	1.79 (1.22–2.52)	1.20 (0.91–1.55)	1.09 (0.78–1.47)	0.89 (0.68–1.14)
**Major non-GI extracranial bleeding** ^**a**^							
Number with bleeds	167	25	9	29	52	28	53
Total person-years	13,048.9	2,057.0	1,409.6	1,638.6	4,594.0	3,589.5	6,522.7
Rate of bleed (per 100 person-years) ^b^	1.28 (1.11–1.49)	1.21 (0.80–1.18)	0.64 (0.31–1.15)	1.77 (1.22–2.52)	1.11 (0.86–1.48)	0.78 (0.53–1.11)	0.81 (0.60–1.04)
**Major intracranial bleeding**							
Number with bleeds	138	19	6	11	16	27	33
Total person-years	13156.4	2073.4	1414.6	1647.9	4621.8	3589.8	6649.1
Rate of bleed (per 100 person-years) ^b^	1.05 (0.88–1.91)	0.92 (0.55–1.43)	0.42 (0.16–0.92)	0.67 (0.33–1.19)	0.35 (0.20–0.56)	0.75 (0.50–1.10)	0.50 (0.34–0.70)
**Any major bleeding (GIB, other extracranial and intracranial bleeding)**							
Number with bleeds	499	80	30	69	119	91	139
Total person-years	12978.1	2042.9	1402.1	1615.3	4560.0	3559.4	6591.7
Rate of bleed (per 100 person-years) ^b^	3.84 (3.51–4.19)	3.92 (3.12–4.83)	2.14 (1.46–3.00)	4.27 (3.33–5.36)	2.61 (2.17–3.10)	2.56 (2.07–3.12)	2.11 (1.78–2.48)

^a^Non-GI extracranial bleeding includes vitreous, urogenital, hemoperitoneal and unspecified bleeding as well as hemoarthrosis, hemopericardium, hemoptysis, hematuria and post-bleeding anemia.

^b^Incidence rate estimates are followed by exact Poisson 95% confidence intervals.

### Logistic-LASSO and logistic-adaLASSO prediction models

The ORs of the selected predictors for the warfarin, DOAC and OAC models assessing GIB, NGIB and MB under the logistic-LASSO and logistic-adaLASSO regressions are presented in S7 and S8 Tables, respectively. The models for GIB, NGIB and MB had concordance statistics ranging from 0.60 (95% CI 0.58–0.62) to 0.66 (95% CI 0.63–0.70) with no statistically significant difference between logistic-LASSO and logistic-adaLASSO models (S7 and S8 Tables, S2 Fig). All models were adequately calibrated (Hosmer Lemeshow test: p>0.05) except for the logistic-LASSO selected OAC model for NGIB (S7 and S8 Tables, S1 Fig). There was little difference in discrimination or calibration between logistic-LASSO selected models and their logistic-adaLASSO counterparts. This was the case for all treatment groups and outcomes (S7 and S8 Tables, S1 and S2 Figs).

With the exception of NGIB, the predictors of each bleeding outcome were similar between the DOAC and warfarin treatment groups. Since the logistic-LASSO MB model derived from OAC user data selected marginally less variables than the logistic-adaLASSO MB model and the performance of the models did not differ significantly across methods, we chose the former as the final model fit. The most important MB predictors in our final MB model were liver disease (OR = 1.64), MB history (OR = 1.57), age ≥ 75 vs < 75 (OR = 1.37) antiplatelet use (OR = 1.28), cardiomyopathy (OR = 1.22), PVD (OR = 1.21) and COPD (OR = 1.21).

The selected model had a c-statistic of 0.63 (95% CI 0.61–0.65) and was well-calibrated (Table 3). The formula representing this model can be seen in Table 3. The final MB model performed just as well in detecting GIB and NGIB as it did for MB (GIB c-statistic: 0.65, 95% CI 0.63–0.66; NGIB c-statistic: 0.67, 95% CI 0.64–0.70; Table 3). However, with regards to calibration, the model underpredicted GIB and NGIB among patients at moderate and high risk of each respective MB subtype (see S3 Fig). To understand how to apply and interpret the selected model, you may refer to the formula for the risk of major bleeding in the year following OAC initiation derived for any OAC new user with AF (Table 3).

**Table 3:** The predictors selected into the primary prediction model of major bleeding and its performance.

	Model coefficients	Model ORs
**Sociodemographic criteria at cohort entry**		
Age ≥ 75 years (ref. <75 years)	0.31	1.37
Female sex	0.08	1.09
**Co-morbidities within 3 years before cohort entry**		
Liver disease	0.49	1.64
History of major bleeding	0.45	1.57
Cardiomyopathy	0.2	1.22
Peripheral vascular (arterial) disease	0.2	1.21
Hypertension	0.14	1.15
Congestive heart failure	0.12	1.14
Chronic obstructive pulmonary disease/asthma	0.12	1.13
Valvular heart disease	0.10	1.10
Acute myocardial infarction	0.09	1.09
Coronary artery disease (excl. MI)	0	-
Other dysrhythmias	0	-
Stroke/TIA	0	-
Dyslipidemia	0	-
Chronic renal failure	0	-
Chronic renal failure ≤ 30 mL/min	0	-
Acute renal failure	0	-
Infection by Helicobacter pylori	0	-
**Concomitant medication use within 2 weeks before cohort entry**		
Antiplatelet	0.25	1.28
Antidiabetics	0.17	1.19
Antidepressants	0.10	1.10
Statin	0	-
NSAIDs	0	-
Proton pump inhibitors	0	-
**OAC type at cohort entry (ref. warfarin)**		
OAC type (apixaban)	-0.37	0.69
OAC type (rivaroxaban)	0	-
OAC type (dabigatran)	0	-
**Model statistics (MB)**		
Cross-val. C-Statistic (95% CI)	N/A	0.63 (0.60–0.65)
Hosmer-Lemeshow test (p-value)	N/A	p>0.05
**Model sensitivity (GIB)**		
Cross-val. C-Statistic (95% CI)	N/A	0.65 (0.63–0.66)
Hosmer-Lemeshow test (p-value)	N/A	p<0.001
**Model sensitivity (NGIB)**		
Cross-val. C-Statistic (95% CI)	N/A	0.67 (0.64–0.70)
Hosmer-Lemeshow test (p-value)	N/A	0.01<p<0.05

The risk of major bleeding in the year following oral anticoagulant initiation as defined by the prediction model derived from a population of all oral anticoagulant users with atrial fibrillation using logistic-LASSO regression can be estimated with $\frac{e^{x}}{1+e^{x}}$ where *x* = -4.51 + 0.31*age_75_and_more + 0.08*is_female + 0.49*liver_disease + 0.45*prior_major_bleeding + 0.2*cardiomyopathy + 0.2*peripheral_vascular_disease + 0.14*hypertension + 0.12*heart_failure + 0.12*chronic_obstructive_pulmonary_disorder_or_asthma + 0.10*valvular_heart_disease + 0.09*myocardial_infarction + 0.25*antiplatelets + 0.17*antidiabetics + 0.10*antidepressants– 0.37*apixaban.

## Discussion

Our study is the first to derive prediction models for MB and MB subtypes from a cohort of DOAC and warfarin new users with AF. It did so using a robust statistical prediction tool. Our MB and MB subtype models were well-calibrated and performed similarly to previously published MB scores. Warfarin and DOAC users presented similar predictors of MB and GIB, not NGIB. This was likely due to the variable locations of bleeding included in the definition of NGIB. We then built a final MB model derived from data from all OAC users. Due to the marginally superior discrimination of the OAC model relative to the warfarin model, it was deemed that the OAC model was more useful than having separate models for DOAC and warfarin users. The most important MB predictors in our final MB model were liver disease, MB history, age≥75, antiplatelet use, cardiomyopathy, PVD and COPD with ORs ranging from 1.21 to 1.64. Notably, the selection of apixaban as a protective factor (OR = 0.69) relative to warfarin corroborates previous observational studies [57, 58]. These findings may be attributable to the superior bleeding profile of apixaban relative to warfarin.

The OR values for the most important predictors of our final model were largely similar to those reported in the analyses used to derive existing MB scores. For the ABS, the population had a similar stroke risk, but was younger (mean age ranging from 68.1 to 73.7) and less at risk of bleeding (mean HAS-BLED ranging from 2.1 to 2.8). The ABS score, which, like us, was derived from OAC users, selected analogous predictors to our model, including prior MB (HR = 1.27, 95% CI 1.18–1.36), antiplatelet therapy (HR = 1.25, 95% CI 1.16–1.35), and COPD (HR = 1.21, 95% CI 1.13–1.30). The most important difference between our model and the ABS score is their selection of CKD. This difference is most likely due to the continuous definition of age given the association between our age categories, kidney function as well as OAC prescription guidelines.

Furthermore, the ORBIT-AF population had a similar age to ours, but a higher stroke risk (a median CHA_2_DS_2_-VASC ranging from 4.0 to 5.0) and lower bleeding risk (a median HAS-BLED of 2.0). The analyses used to create the ORBIT-AF score used warfarin and dabigatran user data, provided similar point estimates and predictors such as age≥75 (HR = 1.38, 95% CI 1.17–1.61), any prior bleeding excluding NGIB (HR = 1.73, 95% CI 1.34–2.23), and antiplatelet therapy (HR = 1.51, 95% CI 1.30–1.75). Like with the ABS score, the selection of CKD is a major distinction to our model. This may be due to their prediction method, the omission of NGIB in the MB history definition or the lower bleeding risk of the derivation cohort.

On the other hand, for each existing MB score, we found differences between some of their OR values and our own. Most notably, the HAS-BLED study presented a significantly different OR estimate for prior MB (OR = 7.51, 95% CI 3.00–18.78), while all other models selected CKD and omitted liver disease. The CKD discrepancy is most likely due to the contraindication of DOAC use among patients with renal dysfunction in our cohort. Moreover, the high prior MB point estimate may be attributable to the small sample size or selection bias attributable to the substantial missing data. However, despite these differences to our model, the HAS-BLED similarly incorporated age≥65 (OR = 2.66, 95% CI 1.33–5.32). Given that the HAS-BLED was derived from warfarin data, it may exclude important MB predictors among DOAC users, hence the need for a score that is derived from a cohort encompassing all types of OAC users.

Our model performed similarly to other MB scores in the literature with a c-statistic of 0.63 (0.60–0.65) and had adequate calibration. The HAS-BLED, (c-statistic: 0.65 [0.61‐0.69]) performed better than existing scores in a meta-analysis of observational studies (c-statistics of 0.63 (0.61‐0.66) and 0.63 (0.56‐0.72) for HEMORR_2_AGES and ATRIA, respectively) with Net Reclassification and Integrated Discrimination Improvement values exceeding 7% (p<0.001) [24, 59–62]. However, unlike our model, few of the studies used cross-validation or bootstrapping to evaluate model performance, which may have led to overconfident assessments if the models were not independently validated [24, 59–63]. Although our model performed similarly to the HAS-BLED, we evaluated its discrimination more robustly and the HAS-BLED was inadequately calibrated [64]. MB prediction scores, such as the ORBIT score and the ABS, which included DOAC user data in their derivation cohort, have performed similarly or slightly better than our model with c-statistics of 0.65 (0.64–0.66) and 0.68 (0.67–0.69), respectively [27, 28].

Our study was one of the few to have tested the ability of its MB prediction model to detect MB subtypes. A real-world study compared the HAS-BLED’s ability to discriminate MB subtypes to that of the Age Biomarker Clinical history score and found that the HAS-BLED performed better in detecting MB (c-statistics: 0.583 and 0.518, respectively) and GIB (c-statistics: 0.596 and 0.519, respectively) [65]. However, these findings were neither cross-validated, nor externally validated [60, 65]. Our own MB risk score overperformed relative to the HAS-BLED in this study (c-statistic: 0.65 95% CI 0.63–0.66), but further research is needed for confirmation. Furthermore, while the HAS-BLED outperformed other scores in predicting ICH, we were unable to evaluate this outcome due to a paucity of events-per-predictors [60, 65]. Finally, despite encompassing approximately half of MB cases, NGIB, which predominantly included genitourinary bleeding and gross hematuria, has been poorly studied [66–68]. Our model predicted NGIB as well as it did MB (c-statistic: 0.67 95% CI 0.64–0.70). Thus, one of the advantages of our MB model is that it also had a good discrimination in terms of GIB and NGIB. Nonetheless, these findings need to be validated with inpatient data.

Furthermore, no study has identified the predictors for the most prevalent MB subtypes among DOAC and warfarin users. Two prediction schemes (the Qbleed models) and one observational study evaluated predictors of upper GIB and ICH as well as all GIB, respectively. However, neither model accounted for all DOAC users [69, 70]. Our study is the first to identify predictors of GIB and NGIB using a derivation cohort of DOAC and warfarin users. Our final model identified similar predictors to existing MB scores, but may be more robust. Clinical scores that effectively predict common MB subtypes like GIB are essential as they can significantly impact patient quality of life, DOAC adherence, and mortality [29, 71].

Our study has several advantages. Firstly, it is the only study to have developed MB and MB subtype prediction models derived from DOAC and warfarin user data. Secondly, this is one of the few studies to calculate cumulative incidence of MB, GIB, ICH and NGIB stratified by dosage for all DOACs. Thirdly, we used a prediction method that minimized the likelihood of overfitting the regression to its derivation dataset, theoretically leading to a more robust model than existing ones [24, 27, 28, 60–62, 64, 72]. Fourth, unlike previous studies, our model’s performance indices have been cross-validated to avoid inflated c-statistics [24, 27, 60–62, 64, 72]. Fifth, we used a dataset large enough to establish models in each treatment subgroup. Sixth, our predictor candidates were well-defined and clinically useful (non-redundant) variables with externally validated coding algorithms. Moreover, we made sure that our outcome definitions were consistent with previous claims-based observational studies. Seventh, patient loss-to-follow-up (mainly death), OAC non-adherence and OAC switching during follow-up could limit model performance. However, our sensitivity analyses suggested that none of these factors have hindered model performance (S9 Table). Ultimately, the observational nature of our data allowed us to characterize real-world predictors of our outcomes.

Our findings presented some limitations. Firstly, prediction modelling is not designed for causal inference, thereby precluding conclusions regarding the impacts of hypothetical interventions on the risk factors. Secondly, due to the nature of our prediction models, these findings are not directly generalizable to any other common OAC indications or edoxaban users. Thirdly, important candidate predictors may not have been evaluated in our models. Specifically, our source data does not include information on alcohol use, tobacco use, ethnicity, over-the-counter aspirin use or labile INR (factors highly associated with bleeding) [24, 73, 74]. Despite the large populational data source, our sample size constrained our ability to identify ICH predictors. Fourth, some patients with prior cardiovascular diseases may not have been identified due to errors in diagnostic coding. Fifth, medication dispensation does not necessarily amount to medication use, resulting in a potential misclassification bias in our cumulative incidence findings and prediction error in our prediction model. Sixth, given our use of real-world data, our findings require external validation using inpatient data [28]. Seventh, our comparisons to published MB models were only speculative given the differences in MB and predictor definitions between models derived from administrative claims data and those derived from inpatient data. Lastly, given our selection of patients who were hospitalized, it is likely that our cohort was older, sicker and used more medications than the general population of anticoagulant users with AF. External validation will be required to ensure the generalizability of our findings to this population.

Our findings have several implications. Due to the overall similarity of MB predictors across treatment groups, our findings suggest that it would be ideal to create an MB risk score that groups together all OAC users rather than generating separate scores for DOACs and warfarin. Moreover, the paucity of RCT and observational data pertaining to GIB and NGIB predictors within an AF population of OAC users makes it difficult to assess whether existing prediction models, such as the HAS-BLED takes into account risk factors for the most prevalent MB subtypes in a real-world population. Thus, although it requires further validation using clinical data and real-world data from other AF patient populations, this study may inform the development of a much-needed monitoring tool that encompasses a more diverse range of MB risk factors adapted to the heterogeneity of OAC user and MB subtype characteristics. Ultimately, our derivation model is well-calibrated and has a similar discriminative potential relative to the other MB scores in the literature (most notably, the HAS-BLED, ABS, and ORBIT-AF), but will require further validation. Future studies will involve using inpatient data to compare our model to the HAS-BLED using adequate comparative performance metrics and seeing how well it stratifies the risk for each MB subtype relative to the HAS-BLED.

## Supporting information

S1 Fig Calibration plots. Calibration plots of LASSO (red) and adaptive LASSO (blue) logistic regression models for GIB among users of A) Warfarin, B) DOACs, C) all OACs; NGIB among users of D) Warfarin, E) DOACs, F) all OACs; and MB among users of G) Warfarin, H) DOACs, I) all OACs.
(TIF)

S2 Fig Cross-validated ROC curves. Cross-validated ROC curves of LASSO (red) and adaptive LASSO (blue) logistic regression models for GIB among users of A) Warfarin, B) DOACs, C) all OACs; NGIB among users of D) Warfarin, E) DOACs, F) all OACs; and MB among users of G) Warfarin, H) DOACs, I) all OACs.
(TIF)

S3 Fig Calibration of plots of the global MB model tested for MB subtypes. Calibration plots of the global MB model tested for its ability to predict A. GIB and B. NGIB.
(TIF)

S1 Table Major bleeding outcome definition. ICD-9 and ICD-10 codes for GIB, NGIB, MB. ICD-9 and ICD-10 codes for GIB, NGIB, ICH and MB. These outcomes were defined on the basis of 6 observational studies [39, 41–45].
(DOCX)

S2 Table Definition of CHADS_2_-VASc_2_, modified HAS-BLED, ATRIA, HEMORR₂HAGES and ORBIT-AF risk scores along with their scoring algorithms. (DOCX)

S3 Table Definition of co-morbidity and concomitant medication variables used for CHA_2_DS_2_-VASc and HAS-BLED risk score calculation according to ICD-9 and ICD-10 codes from the Med-Echo databases. (DOCX)

S4 Table Sample size justification. Assuming 28 candidate predictors, these are the event requirements for each subgroup. ^a^ The number of outcomes in these groups would be sufficient to yield robust prediction models. ^b^ In a simulation study, it was found that under the assumption that outcomes are rare and that noise predictors (predictors presenting redundant information) are present, LASSO regression was shown to yield stable predictions (neither overfitted, nor underfitted models) with an events per candidate predictor ratio of 5.
(DOCX)

S5 Table Baseline characteristics of OAC new user with specific types of major bleeds in the year of follow-up from 2011 to 2018. ^a^ Non-GI extracranial major bleeding as an outcome or a predictor includes vitreous, urogenital, hemoperitoneal and unspecified major bleeding as well as hemoarthrosis, hemopericardium, hemoptysis, hematuria and post-bleeding anemia. All major bleedings included GI, Non-GI extracranial major bleeding and intracranial bleeding. ^b^ DOAC users include all doses of dabigatran, rivaroxaban and apixaban. ^c^ OAC users include all doses of warfarin, dabigatran, rivaroxaban and apixaban. ^d^ Represents a history of at least one of the bleeding subcategories OR at least one prescription of antiplatelet subcategory. Although each subcategory is mutually exclusive, the totals will not add up to the parent variable.
(DOCX)

S6 Table Baseline characteristics of OAC new users without specific types of major bleeds in the year of follow-up from 2011 to 2018. ^a^ Non-GI extracranial major bleeding as an outcome or a predictor includes vitreous, urogenital, hemoperitoneal and unspecified major bleeding as well as hemoarthrosis, hemopericardium, hemoptysis, hematuria and post-bleeding anemia. ^b^ DOAC users include all doses of dabigatran, rivaroxaban and apixaban. ^c^ OAC users include all doses of warfarin, dabigatran, rivaroxaban and apixaban. ^d^ Represents a history of at least one of the bleeding subcategories OR at least one prescription of antiplatelet subcategory. Although each subcategory is mutually exclusive, the totals will not add up to the parent variable.
(DOCX)

S7 Table Logistic regression LASSO analyses of major bleeding subtype predictors among OAC new users from 2011 to 2018. All values are ORs. ^a^ In the DOAC group, the rivaroxaban and apixaban variables are compared to dabigatran. In the OAC group, dabigatran, rivaroxaban and apixaban are compared to warfarin. ^b^ DOAC users include all doses of dabigatran, rivaroxaban and apixaban. ^c^ OAC users include all doses of warfarin, dabigatran, rivaroxaban and apixaban.
(DOCX)

S8 Table Logistic regression adaptive LASSO analyses of major bleeding subtype predictors among OAC new users from 2011 to 2018. All values are ORs. ^a^ In the DOAC group, the rivaroxaban and apixaban variables are compared to dabigatran. In the OAC group, dabigatran, rivaroxaban and apixaban are compared to warfarin. ^b^ DOAC users include all doses of dabigatran, rivaroxaban and apixaban. ^c^ OAC users include all doses of warfarin, dabigatran, rivaroxaban and apixaban.
(DOCX)

S9 Table Sensitivity analyses of the global MB model for all OAC users. Discrimination values for the global score in patients who did not die during follow-up, adherent patients (PDC≥0.80), non-adherent patients (PDC<0.80), patients who did not switch OAC in the year of follow-up and patients who did not switch OAC or die during follow-up.
(DOCX)
