# Supplementary figures and images for: Predicting major bleeding among hospitalized patients using oral anticoagulants for atrial fibrillation after discharge
Source: PLoS One. 2021 Mar 3;16(3):e0246691. doi: 10.1371/journal.pone.0246691 (PMC7928472; doi:10.1371/journal.pone.0246691)

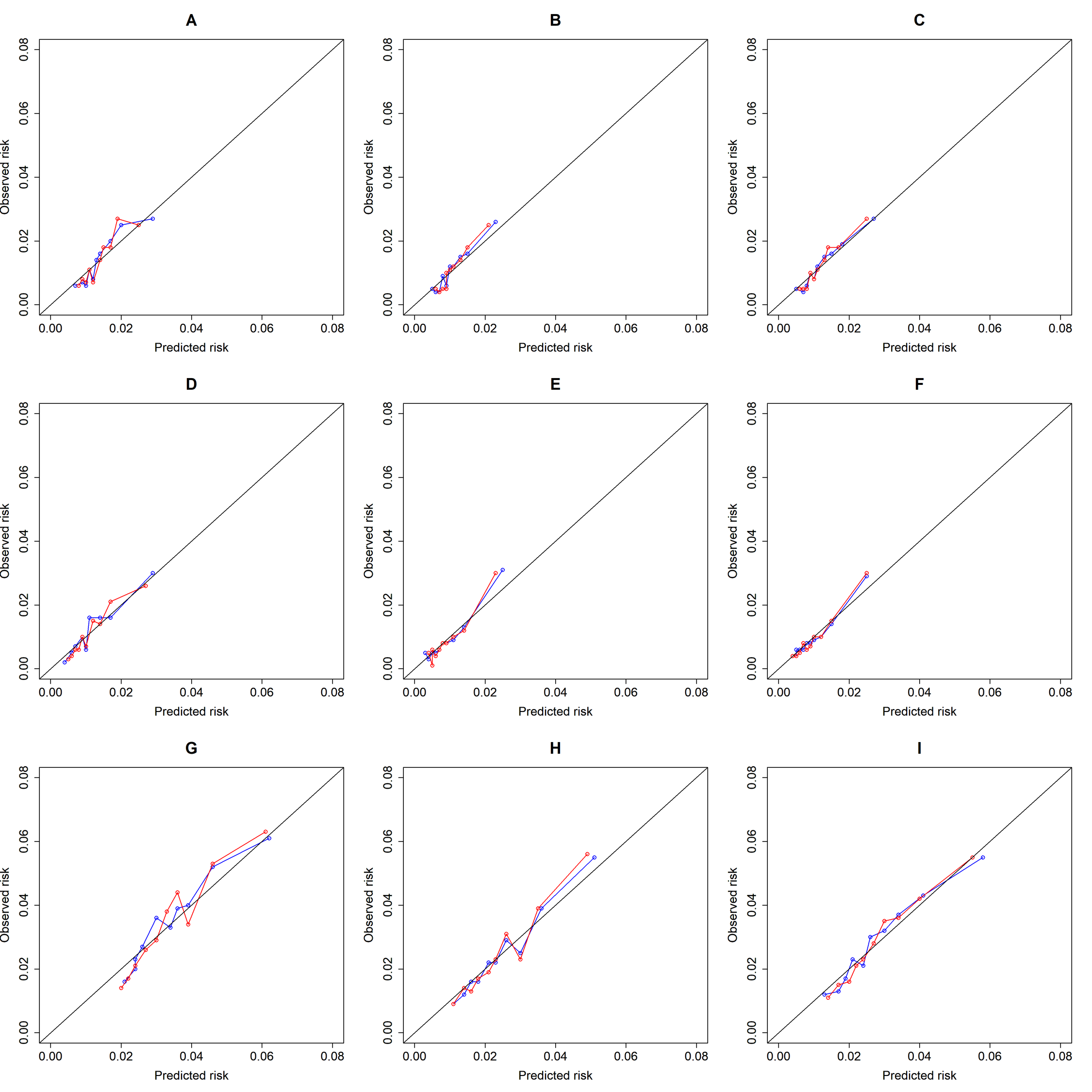

Supplement: S1 Fig — Calibration plots of LASSO (red) and adaptive LASSO (blue) logistic regression models for GIB among users of A) Warfarin, B) DOACs, C) all OACs; NGIB among users of D) Warfarin, E) DOACs, F) all OACs; and MB among users of G) Warfarin, H) DOACs, I) all OACs. (TIF) [file pone.0246691.s001.tif]

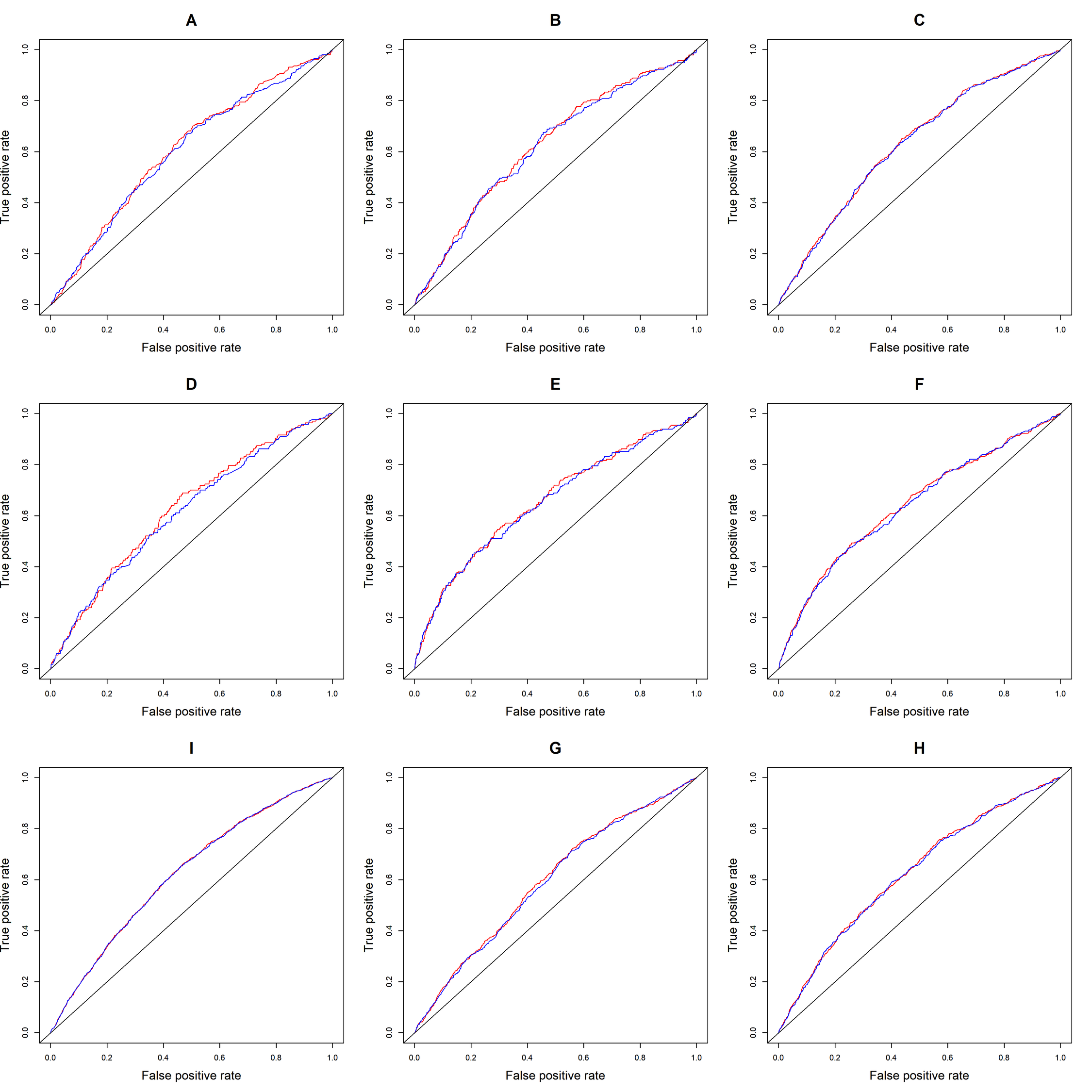

Supplement: S2 Fig — Cross-validated ROC curves of LASSO (red) and adaptive LASSO (blue) logistic regression models for GIB among users of A) Warfarin, B) DOACs, C) all OACs; NGIB among users of D) Warfarin, E) DOACs, F) all OACs; and MB among users of G) Warfarin, H) DOACs, I) all OACs. (TIF) [file pone.0246691.s002.tif]

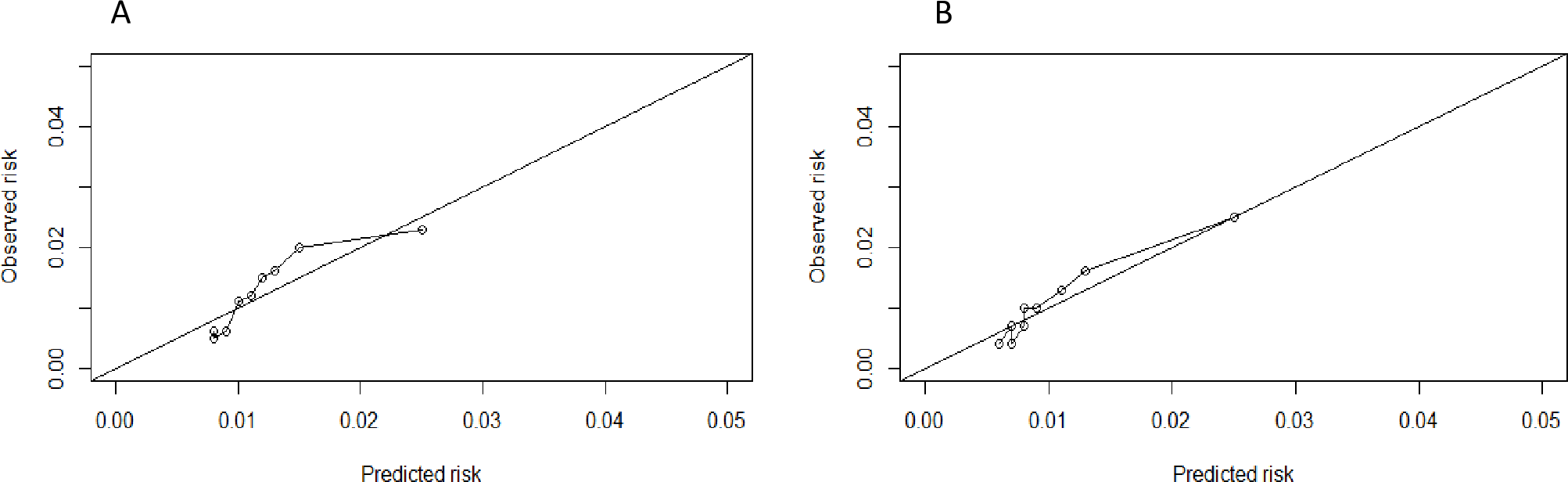

Supplement: S3 Fig — Calibration plots of the global MB model tested for its ability to predict A. GIB and B. NGIB. (TIF) [file pone.0246691.s003.tif]
