# Supplementary material for: Predicting major bleeding among hospitalized patients using oral anticoagulants for atrial fibrillation after discharge
Source: PLoS One. 2021 Mar 3;16(3):e0246691. doi: 10.1371/journal.pone.0246691 (PMC7928472; doi:10.1371/journal.pone.0246691)
Supplement: S1 Table — ICD-9 and ICD-10 codes for GIB, NGIB, MB. ICD-9 and ICD-10 codes for GIB, NGIB, ICH and MB. These outcomes were defined on the basis of 6 observational studies [39, 41–45]. (DOCX) [file pone.0246691.s004.docx]

**S1 Table.** Major bleeding outcome definition.

|  | **ICD-9 codes** | **ICD-10 codes** |
| --- | --- | --- |
| **Major Bleed (MB)** | | |
| **Haemorrhagic stroke intracranial (non-traumatic; ICH)** | 430, 431, 432.x | I60, I61, I62 |
| **Haemorrhagic stroke intracranial (traumatic; ICH)** | 852x, 853x | S063, S064, S065, S066 |
| **Major GI bleeding (GIB)** | Upper GI: 456.1, 530.7, 531.0x, 531.2x, 531.4x, 531.6x, 532.0x, 532.2x, 532.4x. 532.6x, 533.0x. 533.2x, 533.4x, 533.6x, 534.0x, 534.2x, 534.4x, 534.6x, 535.01, 537.83, 578.0  Lower GI: 562.02, 562.03, 562.12, 562.13, 569.3x, 569.85, 578.1x, 578.9 | Upper GI: I850, K226, K250, K252, K254, K256, K260, K262, K264, K266, K270, K272, K274, K276, K280, K282, K284, K286, K2901, K290, K31811, K920  Lower GI: K921, K922, K5711, K5713, K5731, K5733, K625, K5521 |
| **Major Non-GI Extracranial Bleed (NGIB)** | Hematuria: 599.7  Hemoptysis: 786.3x  Vitreous bleeding: 379.23  Urogenital bleeding: 626.2x,280.0 285.1,285.9  Hemarthrosis: 719.1x  Hemopericardium: 423.0x  Hemoperitoneal MB: 568.8  Unspecified MB: 459.0x  Post-bleed anemia: 285.1x | Hematuria: R31  Hemoptysis: R042, R0489, R049  Vitreous bleeding: H43.13  Urogenital bleeding: N92.0, D50.0, D62, D64.9  Hemarthrosis: M250x  Hemopericardium: I31.2  Hemoperitoneal MB: K66.1  Unspecified MB: R58.0  Post-bleed anemia: D62 |

ICD-9 and ICD-10 codes for GIB, NGIB, ICH and MB. These outcomes were defined on the basis of 6 observational studies [1-6].

1. Villines TC,Schnee J,Fraeman K,Siu K,Reynolds MW,Collins J, Schwartzman E. A comparison of the safety and effectiveness of dabigatran and warfarin in non-valvular atrial fibrillation patients in a large healthcare system. Thromb Haemost. 2015; 114: 1290-1298.

2. Yao X,Abraham NS,Sangaralingham LR,Bellolio MF,McBane RD,Shah ND, Noseworthy PA. Effectiveness and safety of dabigatran, rivaroxaban, and apixaban versus warfarin in nonvalvular atrial fibrillation. J Am Heart Assoc. 2016; 5.

3. Lauffenburger JC,Farley JF,Gehi AK,Rhoney DH,Brookhart MA, Fang G. Effectiveness and safety of dabigatran and warfarin in real-world us patients with non-valvular atrial fibrillation: A retrospective cohort study. J Am Heart Assoc. 2015;

4. Maura G,Blotière PO,Bouillon K,Billionnet C,Ricordeau P,Alla F, Zureik M. Comparison of the short-term risk of bleeding and arterial thromboembolic events in nonvalvular atrial fibrillation patients newly treated with dabigatran or rivaroxaban versus vitamin k antagonists: A french nationwide propensity-matched cohort study. Circulation. 2015; 132: 1252-1260.

5. Graham DJ,Reichman ME,Wernecke M,Zhang R,Southworth MR,Levenson M,Sheu TC,Mott K,Goulding MR,Houstoun M,MaCurdy TE,Worrall C, Kelman JA. Cardiovascular, bleeding, and mortality risks in elderly medicare patients treated with dabigatran or warfarin for nonvalvular atrial fibrillation. Circulation. 2015; 131: 157-164.

6. Outcomes of dabigatran and warfarin for atrial fibrillation in contemporary practice. Annals of internal medicine. 2017; 167: 845-854.
