# Supplementary material for: Predicting major bleeding among hospitalized patients using oral anticoagulants for atrial fibrillation after discharge
Source: PLoS One. 2021 Mar 3;16(3):e0246691. doi: 10.1371/journal.pone.0246691 (PMC7928472; doi:10.1371/journal.pone.0246691)
Supplement: S2 Table — (DOCX) [file pone.0246691.s005.docx]

**S2 Table.** Definition of CHA_2_DS_2_-VASc_2_, modified HAS-BLED, ATRIA, HEMORR₂HAGES and ORBIT-AF risk scores along with their scoring algorithms.

| **Risk score definition** | Points, if present |
| --- | --- |
| **CHA_2_DS_2_-VASc stroke risk score** |  |
| Congestive heart failure or left ventricular dysfunction | 1 |
| Hypertension | 1 |
| Age | 1 |
| Age ≥ 75 years | 2 |
| Diabetes Mellitus | 1 |
| Stroke (ischemic stroke, transient ischemic disease or systemic embolism | 2 |
| Vascular disease (myocardial infarction, peripheral arterial disease or aortic plaque | 1 |
| Sex category (female) | 1 |
| **HAS-BLED bleeding risk score** |  |
| Hypertension | 1 |
| Abnormal renal function | 1 |
| Abnormal hepatic function |  |
| Abnormal Stroke (ischemic stroke, transient ischemic disease | 1 |
| Bleeding | 1 |
| Older than > 65 years | 1 |
| Labile 65 – 74 years international normalized ratio (not available) | 1 |
| Drugs (ASA, clopidogrel, prasugrel, ticagrelor, ticlopidine, or non-steroidal anti- inflammatory drugs) in the 1 month preceding the ICH hospitalization or 1month after discharge | 1 |
| Alcohol intake | 1 |
| **ATRIA bleeding risk score** |  |
| Anemia (Male: Hemoglobin <13 g/dL; Female: Hemoglobin <12 g/dL) | 3 |
| Severe Renal Disease (Glomerular filtration rate <30 mL/min or dialysis) | 3 |
| Age ≥ 75 years | 2 |
| Any Prior Hemorrhage Diagnosis | 1 |
| Hypertension History | 1 |
| **HEMORR₂HAGES bleeding risk core** | 1 |
| Hepatic or Renal Disease | 1 |
| Ethanol (Alcohol) Abuse | 1 |
| Malignancy History | 1 |
| Older (Age > 75) | 1 |
| Reduced Platelet Count or Function | 1 |
| Rebleeding Risk (bleeding history) | 1 |
| Hypertension (Uncontrolled) | 1 |
| Anemia (Male: Hemoglobin <13 g/dL; Female: Hemoglobin <12 g/dL) | 1 |
| Genetic Factors (CYP 2C9 single-nucleotide polymorphisms) | 1 |
| Excessive Fall Risk | 1 |
| Stroke History | 1 |
| **ORBIT-AF bleeding risk score** |  |
| Anemia (Male: Hemoglobin <13 g/dL; Female: Hemoglobin <12 g/dL) | 2 |
| Age >74 years | 1 |
| Bleeding history | 2 |
| GFR <60 mL/min/1.73 m2 | 1 |
| Antiplatelet agent use | 1 |
