# Supplementary material for: Predicting major bleeding among hospitalized patients using oral anticoagulants for atrial fibrillation after discharge
Source: PLoS One. 2021 Mar 3;16(3):e0246691. doi: 10.1371/journal.pone.0246691 (PMC7928472; doi:10.1371/journal.pone.0246691)
Supplement: S4 Table — Assuming 28 candidate predictors, these are the event requirements for each subgroup. a The number of outcomes in these groups would be sufficient to yield robust prediction models. b In a simulation study, it was found that under the assumption that outcomes are rare and that noise predictors (predictors presenting redundant information) are present, LASSO regression was shown to yield stable predictions (neither overfitted, nor underfitted models) with an events per candidate predictor ratio of 5. (DOCX) [file pone.0246691.s007.docx]

**S4 Table.** Sample size justification.

|  | **MB** | **GIB** | **NGIB** | **MB** | **GIB** | **NGIB** | **MB** | **GIB** | **NGIB** |
| --- | --- | --- | --- | --- | --- | --- | --- | --- | --- |
|  | **All OACs** | | | **Warfarin** | | | **DOACs** | | |
| **10 events per candidate predictor** | 280^a^ | 280^a^ | 280^a^ | 280 | 280 | 280 | 280 | 280 | 280 |
| **5 events per candidate predictor**^b^ | 140^a^ | 140^a^ | 140^a^ | 140^a^ | 140^a^ | 140^a^ | 140^a^ | 140^a^ | 140^a^ |

Assuming 28 candidate predictors, these are the event requirements for each subgroup. ^a^ The number of outcomes in these groups would be sufficient to yield robust prediction models. ^b^ In a simulation study, it was found that under the assumption that outcomes are rare and that noise predictors (predictors presenting redundant information) are present, LASSO regression was shown to yield stable predictions (neither overfitted, nor underfitted models) with an events per candidate predictor ratio of 5. [7]

7. Pavlou M,Ambler G,Seaman S,De Iorio M, Omar RZ. Review and evaluation of penalised regression methods for risk prediction in low-dimensional data with few events. *Statistics in Medicine*. 2016; **35**: 1159-1177.
