# Supplementary material for: Predicting major bleeding among hospitalized patients using oral anticoagulants for atrial fibrillation after discharge
Source: PLoS One. 2021 Mar 3;16(3):e0246691. doi: 10.1371/journal.pone.0246691 (PMC7928472; doi:10.1371/journal.pone.0246691)
Supplement: S6 Table — a Non-GI extracranial major bleeding as an outcome or a predictor includes vitreous, urogenital, hemoperitoneal and unspecified major bleeding as well as hemoarthrosis, hemopericardium, hemoptysis, hematuria and post-bleeding anemia. b DOAC users include all doses of dabigatran, rivaroxaban and apixaban. c OAC users include all doses of warfarin, dabigatran, rivaroxaban and apixaban. d Represents a history of at least one of the bleeding subcategories OR at least one prescription of antiplatelet subcategory. Although each subcategory is mutually exclusive, the totals will not add up to the parent variable. (DOCX) [file pone.0246691.s009.docx]

**S6 Table.** Baseline characteristics of OAC new users without specific types of major bleeds in the year of follow-up from 2011 to 2018.

|  | **Non GI bleeders** | | | **Non GI extracranial bleeder ^a^** | | | | **-Non major bleeders (all types) ^a^** | | | | | | |
| --- | --- | --- | --- | --- | --- | --- | --- | --- | --- | --- | --- | --- | --- | --- |
|  | Warfarin  (n=14,537) | DOAC ^b^ (n=21,406) | OAC ^c^  (n=35,943) | Warfarin  (n=14,574) | DOAC ^b^  (n=21,444) | OAC ^c^  (n=36,018) | | Warfarin  (n=14,242) | | DOAC ^b^  (n=21,112) | | OAC ^c^  (n=35,354) | | |
| **Sociodemographics** |  |  |  |  |  |  | |  | |  | |  | | |
| Age (mean ± SD) | 80.1 ± 9.2 | 78.2 ± 9.5 | 79.0 ± 9.4 | 80.1 ± 9.2 | 78.2 ± 9.5 | 79.0 ± 9.4 | | 78.9 ± 9.4 | | 78.2 ± 9.5 | | 78.9 ± 9.4 | | |
| Age (%) |  |  |  |  | | |  | |  | |  | | |  |
| ≥ 75 | 73.5% | 64.9% | 68.4% | 73.6% | 64.9% | 68.5% | | 68.3% | | 64.7% | | 68.3% | | |
| Male (%) | 44.6 % | 46.9 % | 46.0 % | 44.5 % | 46.9 % | 45.9 % | | 45.9 % | | 46.9 % | | 45.9 % | | |
| Pampalon index elevated social deprivation (%) | 26.6% | 26.6% | 26.6% | 26.6% | 26.6% | 26.6% | | 26.6% | | 26.6% | | 26.6% | | |
| Pampalon index elevated material deprivation (%) | 25.9% | 25.8% | 25.8% | 25.9% | 25.8% | 25.8% | | 25.8% | | 25.8% | | 25.8% | | |
| **CHA_2_DS_2_-VASc Score** (mean ± SD) | 4.0 ± 1.4 | 3.5 ± 1.4 | 3.7 ± 1.4 | 4.0 ± 1.4 | 3.5 ± 1.4 | 3.7 ± 1.4 | | 3.7 ± 1.4 | | 3.5 ± 1.4 | | 3.7 ± 1.4 | | |
| **CHA_2_DS_2_-VASc Score (%)** |  |  |  |  |  |  | |  | |  | |  | | |
| 0 - 1 | 4.0% | 7.1% | 5.9% | 4.0% | 7.1% | 5.8% | | 5.9% | | 7.2% | | 5.9% | | |
| 2 - 3 | 31.8% | 41.7% | 37.6% | 31.7% | 41.6% | 37.6% | | 37.7% | | 41.7% | | 37.7% | | |
| 4 | 30.8% | 27.9% | 29.1% | 30.8% | 27.9% | 29.1% | | 29.0% | | 27.9% | | 29.0% | | |
| ≥ 5 | 33.5% | 23.4% | 27.5% | 33.5% | 23.4% | 27.5% | | 27.4% | | 23.3% | | 27.4% | | |
| **HAS-BLED score** (mean ± SD) | 3.3 ± 1.3 | 2.9 ± 1.3 | 3.1 ± 1.3 | 3.3 ± 1.3 | 2.9 ± 1.3 | 3.1 ± 1.3 | | 3.1 ± 1.3 | | 2.9 ± 1.3 | | 3.1 ± 1.3 | | |
| **HAS-BLED score (%)** |  |  |  |  |  |  | |  | |  | |  | | |
| < 3 | 26.7% | 39.5% | 34.3% | 26.7% | 39.5% | 34.3% | | 34.5% | | 39.7% | | 34.5% | | |
| ≥ 3 | 73.3% | 60.5% | 65.7% | 73.3% | 60.5% | 65.7% | | 65.5% | | 60.3% | | 65.5% | | |
| **Co-morbidities within 3 years before cohort entry** | | |  |  |  |  | |  | |  | |  | | |
| Hypertension | 84.7% | 79.5% | 81.7% | 84.8% | 79.6% | 81.7% | | 81.6% | | 79.5% | | 81.6% | | |
| Coronary artery disease (excl. MI) | 52.5 % | 48.2 % | 56.2 % | 52.3% | 41.8% | 46.1 % | | 52.5 % | | 41.8 % | | 56.0 % | | |
| Acute myocardial infarction | 15.6 % | 11.2 % | 13.0 % | 12.9 % | 11.2 % | 12.9 % | | 12.9 % | | 11.1 % | | 12.9 % | | |
| Chronic heart failure | 43.9 % | 33.2 % | 37.5 % | 44.0 % | 33.2 % | 37.6 % | | 37.4 % | | 33.1 % | | 37.4 % | | |
| Cardiomyopathy | 6.4 % | 6.1 % | 6.2 % | 6.4 % | 6.0 % | 6.1 % | | 6.2 % | | 6.0 % | | 6.2 % | | |
| Other dysrhythmias | 20.4 % | 19.4 % | 19.8 % | 20.5 % | 19.4 % | 19.8 % | | 19.8 % | | 19.4 % | | 19.8 % | | |
| Valvular heart disease | 22.8 % | 16.1 % | 18.8 % | 22.8 % | 16.1 % | 18.8 % | | 18.7 % | | 16.0 % | | 18.7 % | | |
| Stroke/TIA | 21.0 % | 17.7 % | 19.1 % | 20.9 % | 17.7 % | 19.0 % | | 19.0 % | | 17.7 % | | 19.0 % | | |
| Peripheral vascular (arterial) disease | 25.0 % | 18.4 % | 21.0 % | 24.9 % | 18.3 % | 21.0 % | | 20.9 % | | 18.3 % | | 20.9 % | | |
| Dyslipidemia | 53.8 % | 51.2 % | 52.2 % | 53.7 % | 51.2 % | 52.2 % | | 52.2 % | | 51.2 % | | 52.2 % | | |
| Diabetes | 38.8 % | 32.2 % | 34.9 % | 38.7 % | 32.2 % | 34.8 % | | 34.7 % | | 32.1 % | | 34.7 % | | |
| History of major bleeding ^a,d^ | 32.7 % | 26.8 % | 29.2 % | 32.7 % | 26.8 % | 29.2 % | | 29.0 % | | 26.6 % | | 29.0 % | | |
| History of intracranial bleeding | 3.4 % | 4.2 % | 3.9 % | 3.3 % | 4.2 % | 3.8 % | | 3.8 % | | 4.1 % | | 3.8 % | | |
| History of GI bleeding | 8.0 % | 7.1 % | 7.5 % | 8.1 % | 7.2 % | 7.6 % | | 7.4 % | | 7.0 % | | 7.4 % | | |
| History of other bleeding ^a^ | 25.8 % | 19.3 % | 21.9 % | 25.9 % | 19.2 % | 21.9 % | | 21.8 % | | 19.1 % | | 21.8 % | | |
| Chronic renal failure | 45.4 % | 28.4 % | 35.3 % | 45.4 % | 28.3 % | 35.2 % | | 35.1 % | | 28.3 % | | 35.1 % | | |
| Chronic renal failure ≤ 30 mL/min | 0.9 % | 0.3 % | 0.5 % | 0.9 % | 0.3 % | 0.5 % | | 0.5 % | | 0.3 % | | 0.5 % | | |
| Acute renal failure | 29.4 % | 17.7 % | 22.4 % | 29.3 % | 17.6 % | 22.3 % | | 22.3 % | | 17.6 % | | 22.3 % | | |
| Liver disease | 2.2 % | 2.0 % | 2.1 % | 2.2 % | 2.0 % | 2.1 % | | 2.1 % | | 2.0 % | | 2.1 % | | |
| Chronic obstructive pulmonary disease/asthma | 38.7 % | 35.2 % | 36.6 % | 38.8 % | 35.1 % | 36.6 % | | 36.5 % | | 35.0 % | | 36.5 % | | |
| Infection by Helicobacter pylori | 0.8 % | 0.7 % | 0.7 % | 0.8 % | 0.7 % | 0.7 % | | 0.7 % | | 0.7 % | | 0.7 % | | |
| Depression | 11.3 % | 11.4 % | 11.3 % | 11.3 % | 11.3 % | 11.3 % | | 11.3 % | | 11.3 % | | 11.3 % | | |
| **Concomitant medication use (within 2 weeks before cohort entry) (%)** | | | | | | | |  | |  | | |  | |
| Statin | 47.8 % | 42.9 % | 44.9 % | 47.7 % | 42.8 % | 44.8 % | | 44.7 % | | 42.8 % | | 44.7 % | | |
| All Antiplatelets ^d^ | 35.7 % | 25.7 % | 29.7 % | 35.7 % | 25.7 % | 29.7 % | | 29.6 % | | 25.6 % | | 29.6 % | | |
| Low dose aspirin (ASA) | 31.8 % | 23.0 % | 26.5 % | 31.6 % | 23.0 % | 26.6 % | | 31.8 % | | 22.9 % | | 26.4 % | | |
| Oth. antiplatelets (without ASA) | 6.2 % | 3.8 % | 4.8 % | 6.1 % | 3.9 % | 4.8 % | | 6.1 % | | 3.8 % | | 4.8 % | | |
| Proton pump inhibitors (PPIs) | 49.8 % | 43.3 % | 45.9 % | 49.7 % | 43.2 % | 45.8 % | | 45.8 % | | 43.2 % | | 45.8 % | | |
| NSAIDs | 1.3 % | 1.4 % | 1.4 % | 1.4 % | 1.3 % | 1.4 % | | 1.4 % | | 1.4 % | | 1.4 % | | |
| Digoxin | 13.4 % | 10.4 % | 11.6 % | 13.4 % | 10.4 % | 11.6 % | | 11.6 % | | 10.4 % | | 11.6 % | | |
| Amiodarone | 9.3 % | 8.5 % | 8.8 % | 9.2 % | 8.4 % | 8.7 % | | 8.7 % | | 8.4 % | | 8.7 % | | |
| Antidepressants | 16.6 % | 16.5 % | 16.6 % | 16.6 % | 16.5 % | 16.5 % | | 16.5 % | | 16.5 % | | 16.5 % | | |
| B-Blockers | 62.3 % | 63.4 % | 62.9 % | 62.2 % | 63.4 % | 62.9 % | | 62.9 % | | 63.4 % | | 62.9 % | | |
| Calcium channel blockers | 39.9 % | 35.5 % | 37.3 % | 40.0 % | 35.6 % | 37.4 % | | 37.3 % | | 35.5 % | | 37.3 % | | |
| Inhibitors of renin-angiotensin system | 37.8 % | 36.2 % | 36.9 % | 37.7 % | 36.2 % | 36.8 % | | 36.8 % | | 36.2 % | | 36.8 % | | |
| Diuretics | 44.3 % | 34.6 % | 38.5 % | 44.2 % | 34.6 % | 38.5 % | | 38.4 % | | 34.5 % | | 38.4 % | | |
| Loop diuretics | 37.1 % | 27.4 % | 31.3 % | 37.1 % | 27.4 % | 31.3 % | | 31.2 % | | 27.3 % | | 31.2 % | | |
| Antidiabetics | 23.0 % | 18.8 % | 20.5 % | 22.9 % | 18.8 % | 20.4 % | | 20.4 % | | 18.7 % | | 20.4 % | | |
| **OAC type at cohort entry** |  |  |  |  |  |  | |  | |  | |  | | |
| Warfarin | 100 % | 0 % | 40.4 % | 100 % | 0 % | 40.5 % | | 100 % | | 0 % | | 40.3 % | | |
| Dabigatran 110 mg | NA | 8.9 % | 6.2 % | NA | 10.4 % | 6.2 % | | NA | | 10.4 % | | 6.2 % | | |
| Dabigatran 150 mg | NA | 5.7 % | 4.0 % | NA | 6.9 % | 4.1 % | | NA | | 6.9 % | | 4.1 % | | |
| Rivaroxaban 15 mg | NA | 7.3 % | 5.1 % | NA | 8.4 % | 5.0 % | | NA | | 8.4 % | | 5.0 % | | |
| Rivaroxaban 20 mg | NA | 19.2 % | 13.4 % | NA | 22.5 % | 13.4 % | | NA | | 22.6 % | | 13.5 % | | |
| Apixaban 2.5 mg | NA | 16.3 % | 11.4 % | NA | 19.1 % | 11.4 % | | NA | | 19.1 % | | 11.4 % | | |
| Apixaban 5 mg | NA | 27.9 % | 19.5 % | NA | 32.7 % | 19.5 % | | NA | | 32.8 % | | 19.6 % | | |
| **Charlson score (mean, ± SD)** | 5.0 ± 3.4 | 4.2 ± 3.4 | 4.5 ± 3.4 | 5.0 ± 3.4 | 4.2 ± 3.4 | 4.5 ± 3.4 | | 4.5 ± 3.4 | | 4.2 ± 3.4 | | 4.5 ± 3.4 | | |
| **Charlson score** < 4 (%) | 37.9 % | 50.7 % | 45.5 % | 62.0 % | 50.8 % | 45.6 % | | 45.7 % | | 50.9 % | | 45.7 % | | |
| **Charlson score** ≥ 4 (%) | 71.1 % | 49.3 % | 54.5 % | 38.0 % | 49.2 % | 54.4 % | | 54.3 % | | 49.1 % | | 54.3 % | | |

^a^ Non-GI extracranial major bleeding as an outcome or a predictor includes vitreous, urogenital, hemoperitoneal and unspecified major bleeding as well as hemoarthrosis, hemopericardium, hemoptysis, hematuria and post-bleeding anemia. ^b^ DOAC users include all doses of dabigatran, rivaroxaban and apixaban. ^c^ OAC users include all doses of warfarin, dabigatran, rivaroxaban and apixaban. ^d^ Represents a history of at least one of the bleeding subcategories OR at least one prescription of antiplatelet subcategory. Although each subcategory is mutually exclusive, the totals will not add up to the parent variable.
