# Supplementary material for: Predicting major bleeding among hospitalized patients using oral anticoagulants for atrial fibrillation after discharge
Source: PLoS One. 2021 Mar 3;16(3):e0246691. doi: 10.1371/journal.pone.0246691 (PMC7928472; doi:10.1371/journal.pone.0246691)
Supplement: S7 Table — All values are ORs. a In the DOAC group, the rivaroxaban and apixaban variables are compared to dabigatran. In the OAC group, dabigatran, rivaroxaban and apixaban are compared to warfarin. b DOAC users include all doses of dabigatran, rivaroxaban and apixaban. c OAC users include all doses of warfarin, dabigatran, rivaroxaban and apixaban. (DOCX) [file pone.0246691.s010.docx]

**S7 Table.** Logistic Regression LASSO analyses of Major Bleeding Subtype Predictors among OAC new users from 2011 to 2018.

|  | **GI bleeding** | | | **Non-GI extracranial bleeding** | | | **Major bleeding (all types)** | | | | |
| --- | --- | --- | --- | --- | --- | --- | --- | --- | --- | --- | --- |
|  | Warfarin (n=14,741) | DOAC ^b^ (n=21,640) | OACs ^c^ (n=36,381) | Warfarin (n=14,741) | DOAC ^b^ (n=21,640) | OACs ^c^ (n=36,381) | Warfarin  (n=14,741) | DOAC ^b^ (n=21,640) | | OACs ^c^ (n=36,381) | |
| Age (%) |  |  |  |  |  |  |  |  | |  | |
| ≥75 | 1.13 | 1.42 | 1.39 | - | 1.32 | 1.13 | 1.05 | 1.60 | | 1.37 | |
| Female (%) | - | - | - | 1.20 | - | 1.14 | 1.14 | | - | | 1.09 |
| **Co-morbidities within 3 years before cohort entry** | | | | | | | | | |  | |
| Hypertension | 1.12 | 1.13 | 1.21 | 1.02 | - | 1.08 | 1.14 | 1.09 | | 1.15 | |
| Coronary artery disease (excl. MI) | - | - | - | - | 1.22 | 1.06 | - | 1.02 | | - | |
| Acute myocardial infarction | - | 1.10 | - | 1.43 | 1.25 | 1.41 | 1.04 | 1.11 | | 1.09 | |
| Chronic heart failure | 1.12 | 1.14 | 1.17 | - | - | - | 1.04 | 1.18 | | 1.14 | |
| Cardiomyopathy | - | - | - | 1.36 | 2.15 | 1.86 | 1.04 | 1.26 | | 1.22 | |
| Other dysrhythmias | - | 0.85 | 0.93 | - | - | - | - | 0.99 | | - | |
| Valvular heart disease | - | 1.15 | 1.09 | 1.36 | - | 1.24 | 1.05 | 1.12 | | 1.10 | |
| Stroke/TIA | 0.96 | 0.97 | 0.87 | - | - | - | - | - | | - | |
| Peripheral vascular (arterial) disease | - | 1.01 | 1.07 | 1.20 | 1.21 | 1.26 | 1.15 | 1.23 | | 1.21 | |
| Dyslipidemia | - | 1.02 | - | - | - | - | - | - | | - | |
| History of major bleeding | 1.53 | 1.49 | 1.58 | 1.65 | 1.65 | 1.72 | 1.51 | 1.59 | | 1.57 | |
| Chronic renal failure | - | 0.98 | - | - | 1.21 | 1.12 | - | - | | - | |
| Chronic renal failure ≤ 30 mL/min | - | - | - | 1.08 | - | 1.21 | - | - | | - | |
| Acute renal failure | - | - | - | 1.14 | - | 1.07 | - | - | | - | |
| Liver disease | 1.78 | 2.53 | 2.38 | 1.39 | - | 1.12 | 1.81 | 1.28 | | 1.64 | |
| Chronic obstructive pulmonary disease/asthma | 1.33 | 1.15 | 1.29 | 1.08 | 1.49 | 1.34 | 1.02 | 1.21 | | 1.13 | |
| Infection by Helicobacter pylori | - | - | - | - | 1.20 | 1.29 | - | - | | - | |
| **Concomitant medication use (within 2 weeks before cohort entry) (%)** | | | | | | | | | |  |  |
| Statin | - | - | - | - | - | 1.03 | - | 1.06 | | - | |
| Antiplatelet | 1.27 | 1.16 | 1.29 | 1.23 | 1.02 | 1.16 | 1.37 | 1.12 | | 1.28 | |
| Proton pump inhibitors (PPIs) | - | - | 0.95 | 1.12 | - | 1.07 | - | - | | - | |
| NSAIDs | - | - | - | 0.62 | - | 0.79 | 0.97 | - | | - | |
| Antidepressants | - | - | - | - | - | - | 1.16 | - | | 1.10 | |
| Antidiabetics | - | - | 1.05 | 1.17 | 1.39 | 1.33 | 1.17 | 1.10 | | 1.19 | |
| **OAC used at cohort entry** |  |  |  |  |  |  |  |  | |  | |
| DOAC type (dabigatran) ^a^ | NA | 1.43 | 1.06 | NA | 1.05 | - | NA | 1.47 | | - | |
| DOAC type (rivaroxaban) ^a^ | NA | - | - | NA | 1.52 | 1.28 | NA | - | | - | |
| DOAC type (apixaban) ^a^ | NA | 1.0 (ref) | 0.73 | NA | 1.0 (ref) | 0.71 | NA | 1.0 (ref) | | 0.69 | |
|  |  |  |  |  |  |  |  |  |  |  |  |
| **Model statistics** |  | |  | |  | |  |  |  |  |  |
| Cross-val. C-Statistic (95% CI) | 0.61(0.57-0.64) | 0.63(0.60-0.66) | 0.63(0.61-0.66) | 0.61 (0.57-64) | 0.66(0.62-0.70) | 0.64 (0.61-0.67) | 0.60 (0.58-  0.62) | 0.63 (0.61-0.65) | | 0.63 (0.60-0.65) | |
| Hosmer-Lemeshow p-value | p>0.05 | p>0.05 | p>0.05 | p>0.05 | p>0.05 | 0.01<p<0.05 | p>0.05 | p>0.05 | | p>0.05 | |

All values are ORs. ^a^ In the DOAC group, the rivaroxaban and apixaban variables are compared to dabigatran. In the OAC group, dabigatran, rivaroxaban and apixaban are compared to warfarin. ^b^ DOAC users include all doses of dabigatran, rivaroxaban and apixaban. ^c^ OAC users include all doses of warfarin, dabigatran, rivaroxaban and apixaban.
