# Supplementary material for: Predicting major bleeding among hospitalized patients using oral anticoagulants for atrial fibrillation after discharge
Source: PLoS One. 2021 Mar 3;16(3):e0246691. doi: 10.1371/journal.pone.0246691 (PMC7928472; doi:10.1371/journal.pone.0246691)
Supplement: S9 Table — Discrimination values for the global score in patients who did not die during follow-up, adherent patients (PDC≥0.80), non-adherent patients (PDC<0.80), patients who did not switch OAC in the year of follow-up and patients who did not switch OAC or die during follow-up. (DOCX) [file pone.0246691.s012.docx]

**S9 Table.** Sensitivity analyses of the global MB model for all OAC users.

|  | **Discrimination** (cross-validated c-statistics) |
| --- | --- |
| **Full cohort** | 0.63 (0.61-0.65) |
| **Patients excluding deaths during follow-up** | 0.65 (0.64-0.67) |
| **Adherent patients** (PDC≥0.80) | 0.62 (0.60-0.64) |
| **Non-adherent patients** (PDC<0.80) | 0.63 (0.61-0.65) |
| **OAC switchers during follow-up** | 0.61 (0.57-0.64) |
| **OAC non-switchers during follow-up** | 0.64 (0.62-0.66) |

Discrimination values for the global score in patients who did not die during follow-up, adherent patients (PDC≥0.80), non-adherent patients (PDC<0.80), patients who switched OAC in the year of follow-up and patients who did not switch OAC in the year of follow-up. [7]

7. Pavlou M,Ambler G,Seaman S,De Iorio M, Omar RZ. Review and evaluation of penalised regression methods for risk prediction in low-dimensional data with few events. *Statistics in Medicine*. 2016; **35**: 1159-1177.
